# Integrative systemic and family therapy for social anxiety disorder: Manual and practice in a pilot randomized controlled trial (SOPHO-CBT/ST)

**DOI:** 10.3389/fpsyg.2022.867246

**Published:** 2022-11-04

**Authors:** Christina Hunger-Schoppe, Jochen Schweitzer, Rebecca Hilzinger, Laura Krempel, Laura Deußer, Anja Sander, Hinrich Bents, Johannes Mander, Hans Lieb

**Affiliations:** ^1^Department of Psychology and Psychotherapy, Witten/Herdecke University, Witten, Germany; ^2^Institute of Medical Psychology, Center for Psychosocial Medicine, University Hospital Heidelberg, Heidelberg, Germany; ^3^Helm Stierlin Institute, Heidelberg, Germany; ^4^Department of Clinical Psychology and Psychotherapy, Bergische University Wuppertal, Wuppertal, Germany; ^5^Center for Psychological Psychotherapy, University of Heidelberg, Heidelberg, Germany; ^6^Institute of Medical Biometry, University Hospital Heidelberg, Heidelberg, Germany; ^7^Private Practitioner, Edenkoben, Germany; ^8^Institute of Systemic Training and Development, Weinheim, Germany; ^9^Institute of Behaviour Therapy, Bad Dürkheim, Germany

**Keywords:** integrative systemic and family therapy (ISFT), social anxiety, multi-person, manual, pilot, feasibility, randomized controlled trial, cognitive-behavioral therapy

## Abstract

Social anxiety disorders (SAD) are among the most prevalent mental disorders (lifetime prevalence: 7–12%), with high impact on the life of an affected social system and its individual social system members. We developed a manualized disorder-specific integrative systemic and family therapy (ISFT) for SAD, and evaluated its feasibility in a pilot randomized controlled trial (RCT). The ISFT is inspired by Helm Stierlin’s concept of related individuation developed during the early 1980s, which has since continued to be refined. It integrates solution-focused language, social network diagnostics, and genogram work, as well as resource- and problem orientation for both case conceptualization and therapy planning. Post-Milan symptom prescription to fluidize the presented symptoms is one of the core interventions in the ISFT. Theoretically, the IFST is grounded in radical constructivism and “Cybern-Ethics,” multi-directional partiality, and a both/and attitude toward a disorder-specific vs. non-disorder-specific therapy approach. SAD is understood from the viewpoint of social systems theory, especially in adaptation to a socio-psycho-biological explanatory model of social anxiety. In a prospective multicenter, assessor-blind pilot RCT, we included 38 clients with SAD (ICD F40.1; Liebowitz Social Anxiety Scale, LSAS-SR > 30): 18 patients participated in the ISFT, and 20 patients in Cognitive Behavioral Therapy (CBT; age: *M* = 36 years, *SD* = 14). Within-group, simple-effect intention-to-treat analyses showed significant reduction in social anxiety (LSAS-SR; ISFT: *d* = 1.67; CBT: *d* = 1.04), while intention-to-treat mixed-design ANOVA demonstrated the advantage of ISFT (*d* = 0.81). Per-protocol analyses supported these results. The remission rate based on blind diagnosticians’ ratings was good to satisfactory (Structured Clinical Interview, SCID; 78% in ST, 45% in CBT, *p* = 0.083); this has yet to be verified in a subsequent confirmatory RCT. The article will present the ISFT rationale and manual, including a special focus on multi-person settings, and the central findings from our pilot RCT.

## Healing as a joint achievement

Collective psychotherapy cultures follow the principle of “healing as a joint achievement” (Schweitzer, 2014). They incorporate an understanding of psychotherapy that has been pushed back in individualized societies to the advantage of individual diagnosis and intervention (“single-person therapy”). Collective psychotherapy cultures, however, have the potential to make a significant difference in the *discourse of psychotherapy* when, in addition to “index clients” and therapists, therapy equally includes family members, friends, colleagues, neighbors, and co-workers, as well as supervisors (“multi-person therapy”). They all can contribute to the development, maintenance, and change of mental disorders and physical illnesses. Therapists who value and believe in the engagement of all these social system members, even if they completely contradict each other, embody core principals of systemic thinking: e.g., radical constructivism and “Cybern-Ethics” (von Förster and Ollrogge, 2008; McNamee and Hosking, 2012), multi-directional partiality and neutrality (Boszormenyi-Nagy and Sparks, 1973; Cecchin, 1987), and a both/and-attitude toward a disorder-specific vs. non-disorder-specific systemic therapy (Lieb, 2009). Characteristics of the *habitus* encompass acting, rehearsal, and playing, in contrast to simply sitting and listening. A flexible composition of the therapy setting is the *norm*, allowing single- to multi-person conversations as well as setting changes.

Therapeutic conversations can include one client or a couple, the family, colleagues, superiors and professionals from various institutions (e.g., the school, youth welfare office), social workers, and doctors. Psychotherapy as cultural practice takes place not only in sacred spaces such as the therapy room, but also in profane places such as the home, in schools, the office, or playgrounds. The location, frequency, duration, and number of therapy sessions vary greatly depending on the clients’ concern and the context of the therapy. In our Heidelberg practice-research group, we developed an Integrative Systemic and Family Therapy (ISFT; Schweitzer et al., 2020). The ISFT includes the therapeutic stance, rationale of disorder and intervention as represented by collective psychotherapy cultures and applies it to social anxiety disorders. It was examined in a feasibility study and showed trends toward positive therapeutic change when compared to Cognitive Behavioral Therapy (CBT; Hunger et al., 2020; Hunger, 2021).

## Therapeutic stance

### Radical constructivism and Cybern-Ethics

The ISFT grounds in radical constructivism (Gadenne, 2008): cognizance emerges within a creative process of constructing various realties (“multiverse”; Tjersland, 1990). The existence of objective facts is not denied, but the epistemological relevance of the world’s ontological representations is challenged (McNamee and Hosking, 2012). Radical constructivism, and with it the idea that every observation essentially depends on and is influenced by the person that makes the observation, builds the epistemological counterpart to second-order cybernetics (Maturana, 1987; Bateson, 2000). Contrarywise, first-order cybernetics is limited to the reciprocity of the different parts, i.e., members, of an affected social system (Selvini Palazzoli et al., 1977).

Every communication and interaction is subject to complexity-reducing observation processes (“perception-taking” [dt. “Wahr-Nehmung”]). On the one hand, our brain selects a few explanations from a wealth of possible explanations: we perceive something. An example: even though the consciously visible spectrum (light) is between 380 and 780 nm, we do not consciously perceive UV or infrared radiation due to our biological condition. Likewise, cultural, social, familial up to individual and (epi-)genetic imprints determine which information we select as significant. On the other hand, it is a matter of meaning-making in (un)conscious reciprocal reaction to what is perceived (“perception-giving” [dt. “Wahr-Gebung”]). Another example: Someone who reacts to a marriage proposal with the answer “yes” gives a social system, e.g. spouse to be, parents-in-law to be, own parents and friends of the couple, a fundamentally different information compared to someone who says “no.” If we follow the reciprocity of “perception-taking” and “perception-giving,” our base of possible beliefs in objective facts thus begins to dodder. Ethical points of view no longer ground on the suchness of the world. What remains is an increasing taking of responsibility on how we encounter and can influence the world around us (“Cybern-Ethics”) (Table 1; von Förster and Ollrogge, 2008).

**Table 1:** Radical constructivism and Cybern-Ethics (von Förster and Ollrogge, 2008; Schweitzer et al., 2020; Hunger, 2021).

**Process qualities**	**Therapeutic consequences**
Perception-taking [dt. “Wahr-Nehmung”]	When we form an opinion about a social system, it is helpful to think that we could always describe it differently. We need to consider whether a description we favor is helpful or whether another description might allow more options for action. Truth (“it is so”) is replaced by the criterion of usefulness (“it is helpful to understand it that way”).
Perception-giving [dt. “Wahr-Gebung”]	When we picture a social system, the probability increases that it behaves accordingly. When we have problems with a social system, we can ask ourselves about our impact on the creation of this “problematic system.” When we get involved with this thinking, it is harder not to take responsibility for how we interact with others: “A disorder is a shared construct!” (Borst, 2017).
Cybern-Ethics	We are in charge of how we perceive and interact with the world around us: every day, it is our decision which reality we opt to live in! We have to give reason whereto we see the world this or that way, and we have to take responsibility for the consequences of our actions!

### Multi-directional partiality (Neutrality)

In the ISFT, the core therapeutic stance embodies multi-directional partiality (neutrality), i.e., the unconditional respect of the (1) *meaningfulness of symptoms*, (2) *ambivalence to (not) change*, and (3) *autonomy* considering the question of who attaches importance to which therapeutic offers. Multi-personal perspectives lean on the phenomenon that social system members primarily follow their own meaning-making, which does not necessarily generate social realities compatible with other social system members. *Construct neutrality* addresses the co-existence of different explanatory models regarding the emergence, maintenance, and change of problems and solutions. The core question is about the purpose of preferring one reality over the other. Loss of construct neutrality is reflected in the unidirectional favoring of a specific explanatory model. At worse, this merely is the explanatory model of the therapists while neglecting the clients’ point of view. *Relational neutrality* occurs when relationship offers are made equally to each social system member and no one is addressed more strongly compared to others, at best while conceding similar speaking times to each social system member. *Problem-solution-neutrality* grounds in the equal validation of change and no-change. It is considered lost, if one holds on to the imperative that the affected social system must change although it still needs time in the problem space, and vice versa. (Loss of) neutrality is not a static event, but a dynamic-interactive process that oscillates in time and requires reciprocal client(s)-therapist(s)-communication. The ISFT therefore uses brief supervisions where, e.g., clients are asked: “As family members, do you experience yourself as equally seen and valued by us therapists, or is there any favouritism?” (Cecchin, 1987; Schweitzer et al., 2020; Hunger, 2021).

### (Non-)Disorder-orientation

In the development and piloting of the ISFT, we often discussed the need for how much, and whether at all, we needed a disorder-orientation. Lieb (2013) discusses four positions and argues for a both/and attitude, which we prefer for the ISFT as well. It includes the unification of the positive aspects of and discourse with experts from other therapeutic positions. We remain neutral toward the symptoms, not labeling them as good or bad, but primarily as meaning-making (Emlein, 2010). We understand disorders as the striving for the best solution at a particular time in the context of a significant transition (Schweitzer et al., 2020). According to Cybern-Ethics (Table 1), diagnoses are understood as a consequence of social negotiation and above all are subject to the question whether they are of use to the affected social system (*utilization principle*; Hammel, 2011). They embody attributions vs. intrasystemic or intraperson truths (Hacking, 1999, 2006). Diagnoses are the link to those who work with clinical codes (e.g., physicians, psychiatrists, psychotherapists, and health insurance companies). In the ISFT, they are used if clients call for them (e.g., “Now this ‘something’ has a name!”), and if they serve the social system (e.g. “The symptoms, i.e., this disorder, is my protective shield against overburdening!”). Nevertheless, the main focus of ISFT still is the exploration and testing of other ways of creating meaning (Schweitzer et al., 2020).

## Conceptualization of social anxiety disorders

### Symptomatology

Social anxiety disorders (SAD) are one of the most prevalent mental disorders (lifetime prevalence: 7–16%), with high impact to those who constitute a social system that includes SAD. The core symptom of SAD is the fear of rejection, ongoing for at least 6 months in one or more social interaction or performance situations while being confronted with unfamiliar people. The social situations are avoided or endured with intense fear or anxiety. SAD is associated with considerable psychosocial handicaps, and increased risk for comorbid disorders and suicidality (Ruscio et al., 2008). Remission rates are low (e.g., 20% in the first 2 years) compared with affective and other anxiety disorders (Yonkers et al., 2003).

### Systemic explanatory model

**Figure 1:**
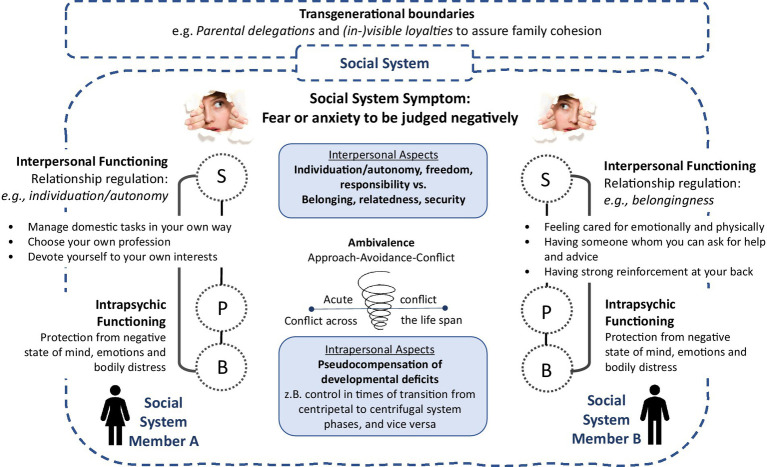
Socio-psycho-biological explanatory model adapted to social anxiety (Luhmann, 2017; Hunger et al., 2018; Schweitzer et al., 2020). S, Social system (verbal and non-verbal communication and interaction); P, Psychological system (thoughts, emotions); and B, Biological system (corporeal parameters).

In the context of the ISFT, we do not understand social anxiety as a mischief and troublemaker but to some degree as reasonable or even useful. It triggers *adaptive self-organization* (e.g., adolescents moving out of parental home; parents focusing on (re)available times), *use of innate behavioral programs* (e.g., endorphin release in times of crisis), and *adaptive modification as well as reorganization of neural networks* (e.g., learning from experience). Social anxiety appears as an existential experience and a component of everyday life (Hüther, 2008), including various aspects of inter- and intrapersonal functioning as depicted in the socio-psycho-biological explanatory model adapted to social anxiety (Luhmann, 2017; Hunger et al., 2018; Figure 1).

In terms of *interpersonal functioning*, we understand social anxiety as an indicator, i.e., the symptom, of unsatisfactory social interactions between at least two social system members often emerging from an unresolved developmental process that affects the whole social system. It may arise as a mismatch of, e.g., one’s sense of belonging while on the other hand striving for the exploration of the world, in need for individuation, or autonomy. Children or adolescents with SAD may thereby ask themselves and others: e.g., “How can I become independent without losing contact to my parents?” (related autonomy); “How do I want to face the world, and as who?” (self-image); and “What friendships do I want to maintain, and how do I want to encounter superiors compared to my parents?” (social contact). Parents can similarly ask themselves and others: e.g., “How can I become more autonomous (again), take advantage of (new) freedoms, and still stay in touch with my descendants?” (related autonomy); “What do I (still) want to experience in the world, and as who?” (self-image); and “What friendships do I want to (re)intensify, and what parts of my personal and/or professional life do I want to expand?” (social contacts). It is precisely these questions, among others, that therapy is concerned with, and which are addressed in the ISFT. The concept of Related Individuation (Stierlin, 1976) follows this idea. Relatedness, and belonging, describe the experience of being an acknowledged member of a social system. This is shown through being respected and welcomed, and of forming and maintaining significant relationships with others in the social system. Belonging is essential to protecting the boundaries of the social system. Individuation describes the standing up for one’s own needs and implies the understanding that rights, responsibilities, appropriate indebtedness, closeness, and distance can be negotiated. Both relatedness and individuation can be understood as two sides of a coin which together are essential for the growth of individual social system members as well as the social system as a whole. A well-balanced Related Individuation allows for the establishment of a new social system, e.g., conjugal family, without having to abandon ties, e.g., regarding one’s own family of origin. In SAD, symptoms serve the prevention of openly communicating desires of freedom which is associated with threatening the existence of the social system. Instead, they weld all social system members together. However, the desire for appropriate distance persists as seen in (non)verbal communication and interaction (e.g., relationship breakdown, aggression). The successful resolution of entangled social relations therefore requires a co-evolutionary process of the social system as a whole, in which all system members are significantly involved.

In terms of *intrapersonal functioning*, we see social anxiety as kind of intrapsychic gain, e.g., when it serves the prevention of negative emotions (e.g., fear and anger) and as pseudo-compensation for developmental deficits of the social system in the transition from a centripetal phase with narrow family ties (e.g., in times of a newborn) to a centrifugal phase with looser family ties (e.g., adolescents’ moving out of home). Social system changes can be frightening when failure seems imminent and with it the threat of the social system’s existence (e.g., breakup of the family of origin). Avoidance of anticipated negative consequences, however, reinforces the idea of existential threats, and thus reciprocally produces what is attempted to be avoided. Spill-over effects of negative communication and interaction patterns, emotions, and thoughts, finally cause a sometimes very pronounced socio-psycho-biological impairment of the various social system members (Priest, 2009).

*Transgenerationally*, significant social system patterns have an impact on the inter- and intrapersonal perception of the social anxiety. Social restraint, e.g., a “keeping one’s head down,” may have been essential for survival in wartime. Parental delegations, e.g., a “make it the same like me as your father/mother” may serve as *(in-)visible loyalties* (Boszormenyi-Nagy and Sparks, 1973; Stierlin, 1976), fostering family cohesion. Every detail of a family biography becomes part of a multi-layered pattern that co-determines the identity of the social system members in the here-now. The past becomes the prologue (Petry and McGoldrick, 2013). However, once the war and/or delegation is over and one steps out of the group, social anxiety prevents the successful mastering of the developmental challenge. It emerges as a maladaptive and repetitive prophylactic power play that unravels along the question of who defines the relationship and how (Selvini Palazzoli et al., 1977; Hand, 2002). This may be the clients with SAD motivating their partners to protect them, who finally take over all activities, above all those outside the home. This can also be the partners who put a stop to the clients’ less trusting behavior, e.g., by interrupting the excessive preparation of a speech and inviting the client as a partner to go for a walk. *Sense-making* becomes the central phenomenon. In terms of meaning-making, one person has to communicate, and another person has to perceive what is communicated as meaningful (e.g., by understanding the utterance, “Please accompany me to psychotherapy!”). The person has to connect (e.g., by agreement or disagreement: “I would never let you leave alone at home!,” or “I think you can manage quite well without me!”; Emlein, 2010). If this sense of reference (*focus*) is missed, disorder-specific symptoms can become the organizing principle of the social systems’ inter- and intrapersonal relations (Luhmann, 2017).

## Treatment of social anxiety

A detailed description of the ISFT can be found in the ISFT manual (Schweitzer et al., 2020). For this paper, we choose those aspects which we experience to be central components of the ISFT but less known in their disorder-specific choreography in the systemic community.

### Manual structure and flexibility

In the ISFT we distinguish four therapy phases with approximately 25 therapy hours, including different objectives with mandatory as well as optional interventions (Table 2, for an overview of the composition of interventions; Table 3, for an overview of the therapy structure). The practice of the ISFT allows for a certain degree of flexibility which seems essential to us when speaking of a client-centered therapy process orientation. The initial phase ideally comprises four sessions with five therapy hours, but in the case of more multi-person conversations it, however, can be fruitful to extend this phase and use therapy hours from other therapy phases such as the mid-phase which, in turn, will then be carried out in a more streamlined way. The same applies when, e.g., the initial phase appears to be finished after three therapy hours, so that the mid-phase begins in the fourth therapy hour. Ideally, the ISFT should be completed in about 22 therapy hours, with three sessions for consolidation after about 6-, 9-, and 12-month post starting the ISFT.

**Table 2:** Objectives depending on the therapy phase, with mandatory as well as optional interventions (Schweitzer et al., 2020).

***Phase***	***Initial phase***	***Mid-phase***	***Final phase***	***Refreshment***
**Objective**	**Development of therapeutic relationship, understanding of the social system and the case, and therapy planning**	**Interventions and integrations**	**Balancing and prolapse prevention**	**Consolidation**
**Content**	Joining; systems diagnostics; construction of goals and contract; and therapy planning	Experimentation with possible changes	Congratulations; dealing with possible prolapses; and ending of therapy	Congratulations; dealing with possible prolapses; and stabilization of successes
**Interventions, mandatory**	Getting to know each other, building confidence Goals and motivation Who is Who: genogram work and social network interview Contextualization, and subjective theories of the social anxiety Journey through time: past, present, and future with respect to the social anxiety (timeline) Shared case construction: good reasons for living with more or less social anxiety Treatment planning: “Who do I/we want to meet and when in therapy, and what do I/we want to do in the here-now as well as in the future?”	Clarify if/why time for change is ready right now, or not yet If no: reframing and symptom prescription If yes: seek out attractive situations and contexts associated with the social anxiety; with therapist, therapy partner, or alone; virtual or real encounter with these events Group session: choral speaking to deconstruct belief systems associated with the social anxiety	Balancing Congratulation, including a person-centred certificate symbolizing therapy success Coping with a life “after social anxiety” as well as after “therapy” as social support system Dealing with future invitations to communication and interaction patterns, thoughts, and feelings associated with social anxiety Farewell to a “life without therapy”	Balancing Congratulations, including the making of distinctions in case of prolapses Evaluation of goal attainment in the here-now Support of successful action in a life “after the social anxiety and without therapist”
**Interventions in all phases, optional**	Observation tasks: When, where, and with whom (no) social anxiety comes and goes? Scaling the intensity of the social anxiety, motivation to change, relationship quality, and social system functioning Solution and aggravation questions Pointing out the smallest signs of change Recognition and positive evaluation of change as well as non-change Adjustments to therapy goal planning in interim evaluations Interventions from all treatment phases can also be used in other therapy phases			

**Table 3:** Course of ISFT in social anxiety disorders, ideal type (Schweitzer et al., 2020) The number of multi-person conversations is perceived as the minimum of sessions where significant social system members are involved. It is welcomed to increase multi-person conversations up to a complete multi-person therapy.

***Phase 1: Initial phase for orientation and therapy planning (approx. 2 months)***
**Session 1: Joining and construction of goals and contract (1 h)** *Joining:* welcome, orientation in the therapy room, introduction of therapist(s) and client(s), above all beyond problems and symptoms *Prompt (problem exploration):* “What problems and symptoms are (not) described? What is their history? In which social systems and contexts do they (not) occur?” *Concern (solution exploration):* “Assuming that the problems and symptoms have resolved one day, how do you (not) live and love? With whom and where (not)?” *Subjective theory:* “How do you explain the problems and symptoms, as well as the solution? Which consequences of action enable or hinder these explanations?” *Contract:* “What does a specific, positive, self-achievable, innovative and attractive solution looks like, that accounts for your living environment?”
**Session 2: Genogram and/or social network interview (1 h)** *Understanding the problems and solutions:* “For which problems do the symptoms appear to represent an attempt at a solution? What sense do they make? Where do they seem to (not) be useful?” *Resources:* “What family and social strengths are evident at the collective as well as the individual level?”
**Session 3: Multi-person conversation (2 h)** *Co-burden and co-treatment:* “To what extent do you, as a multi-person system, experience psychosocial burden? Which prompts, concerns and contracts for co-treatment do you address?” *Support*: “To what extent do you experience yourselves as supportive? What do you want to contribute?” *Patterns of interaction:* “Which (not) successful patterns of previous (not) successful solution attempts can you report?”
**Session 4: Shared case construction and therapy planning (1 h)** *Case construction:* “How does the therapy system, i.e., client(s), therapist(s), and significant other(s), describe problematic inter- and intrapersonal interaction patterns that provoke and maintain the symptoms (“attempts to solutions that have become problems”), their evaluation (“good reasons for staying with more or less of these symptoms”), and their solutions (“attractions to live life in an appropriate living environment”)?” *Therapy goal planning:* selection and prioritization of desired changes along the question of “Who do we/I want to meet and when during the therapy, and what do we/I want to do with and without whom and when?”
***Phase 2: Mid-phase for intervention and integration (approx. 6 months)***
**Session 5: Planning of the 1**^**st**^ **outreach intervention in an approximately mid-level problematic private or professional environment (1 h)** *Acteurs*: client(s), e.g. with peers, colleagues *Conditions to change:* “To what extent is time for change (not) ripe?” *If not*: appreciation of the good reasons for not changing yet; possible interventions: reframing and symptom prescription *If yes*: planning of a concrete outreach intervention in the sense of an attractive and problem-associated situation; (not) accompanied by therapist(s) and/or significant other(s)
**Session 6: Experimentation in client(s)’ daily private or professional environment (2 h)** *Implementation:* “To what extent does the intervention (not) succeed as planned in session 5? Who is (not) involved? Which communication and interaction patterns can (not) be consciously controlled? Who does (not) attend the intervention?”
**Session 7: Multi-person conversation for planning the second outreach intervention in a mid- to high-level problematic private or professional environment (2 h)** *Acteurs*: client(s), e.g. with peers, colleagues *Conditions to change*; *if (not) ready to change:* see session 5 *Implementation:* see session 6
**Session 8: Evaluation of 1st and 2nd outreach intervention; planning of 3**^**rd**^ **outreach intervention (1 h)** *Acteurs*: client(s), with anyone who is of importance, including e.g. strangers *Conditions to change*; *if (not) ready to change:* see session 5
**Session 9: Experimentation in the client(s)’ daily private or professional environment (2 h)** *Implementation:* see session 6
**Session 10: Choral speaking and group conversation (3 h)** *Showing up and learning from other clients:* “How do clients experience themselves and others while exchanging coping experiences and interaction with others who report similar symptoms, in the presence of their therapist(s) and supervisor(s)?” *Choral speaking:* Deconstruction of problem- and symptom-promoting beliefs within a group setting through the singing of the client(s)’ belief systems by the group, and where newly created sentences are converted into new choral parts, until the clients begin to show altered reactions
**Session 11: Evaluation of 1st to 3rd outreach intervention, including the choral speaking session, planning of further (outreach) interventions, if necessary (1 h)**
***Phase 3: Final phase for balancing and prolapse prevention (approx. 4 months)***
**Session12: Balancing of progress and looking ahead to a life without therapy (1 h)** *If therapy progress has been achieved:* congratulations, e.g., “certificate presentation” whereupon the client(s) report about their achievements, including a “recipe” for stabilization (“success work”); planning of further interventions, if necessary *If therapy progress has been little to not achieved:* appreciation of the good reasons for not changing yet; possible interventions: reframing and symptom prescription; planning how to live with the problems and symptoms (“arrangement counselling”)
**Session 13: Multi-person conversation (2 h)** see session 12, in shared conversation with all important social system members
**Session 14: Balancing and stabilization experiments (1 h)** *If therapy progress has been achieved:* “How can therapy progress (not) be stabilized? Who or what can (not) cause it to dodder? Which new conflicts (not) show up? How can future invitations to “honorary rounds in the old pattern” (not) be dealt with successful?” *If therapy progress has been little to not achieved:* see session 12
**Session 15: Balancing and farewell (1 h)** *Farewell to a “life without therapy”:* e.g. giving of stabilization symbols, farewell rituals, final awarding of the “certificate” closing an interview process that has started in about session 12
***Phase 4: Refreshment and consolidation (approx. 3 months)***
**Session 16: Consolidation (1 h), multi-person conversation can, but do not have to, be practiced** *If therapy progress has been achieved and/or stabilized:* “How can therapy progress (not) be consolidated? Who or what can (not) cause it to dodder? Which new conflicts (not) show up? How can future invitations to ‘honorary rounds in the old pattern’ (not) be dealt with successful?” *If therapy progress has been little to not achieved and/or stabilized:* see session 12
**Session 17: Consolidation (1 h), multi-person conversation can, but do not have to, be practiced** *If therapy progress has been achieved and/or stabilized:* “How can therapy progress (not) be consolidated? Who or what can (not) cause it to dodder? Which new conflicts (not) show up? How can future invitations to ‘honorary rounds in the old pattern’ (not) be dealt with successful?” *If therapy progress has been little to not achieved and/or stabilized:* see session 12
**Session 18: Consolidation (1 h), multi-person conversation can, but do not have to, be practiced** *If therapy progress has been achieved and/or stabilized:* “How can therapy progress (not) be consolidated? Who or what can (not) cause it to dodder? Which new conflicts (not) show up? How can future invitations to ‘honorary rounds in the old pattern’ (not) be dealt with successful?” *If therapy progress has been little to not achieved and/or stabilized:* see session 12

Therapy time: 1 h amounts to 50 min. Social system members: they can be part of private social systems (e.g., family, partner, (best)friends and/or professional social systems like e.g., colleagues, superiors).

Multi-person conversations and outreach interventions in the social reality of the client’s daily life can be used as single sessions of 50 min or double sessions of 100 min. The choral speaking is conducted as a group session of 150 min. Other variations are conceivable: e.g., therapy hours can be subdivided and complete a week with a debriefing of 25 min after an outreach intervention of 125 min a few days ago; e.g., several sessions of 25 min each can serve a kind of “therapy break” after therapy goals have been (partly) achieved, and for the stabilization of significant changes.

From our point of view, each manual is an ideal suggestion from which there are good reasons to deviate from in individual cases. It seems optimal to us to *take the ISFT manual sufficiently seriously*, *but not too seriously*. This is the reason why we use bullet points instead of numbering in the description of mandatory and optional interventions (Table 2). All interventions are interchangeable in their order. Even therapy planning in phase 1 can be started in the first therapy hour. In particular, it is important to define with the clients which duration and intensity, for both the single therapy session as well as the intervention, seems most useful under which circumstances.

### Multi-person conversation

The aim of multi-person conversations in the ISFT is to get a shared idea of who is involved in the development, maintenance, and change of the presented problems and symptoms, and who can participate in the therapy process. It is about exploring who has the power to offer optimal *support* as well as to *worsening* the situation. Especially in the initial phase, it seems favorable that the social system members are bound to a secure and safety therapeutic atmosphere that may empower them to give each other their “blessing” in trying out changes. Changes may address distancing movements (e.g., leaving parental home; acceptance of full-time jobs by both parents) as well as approach movements (e.g., trustfulness in family cohesion while announcing a job engagement; becoming an artist, that was not part of the family history yet, e.g., in a family of engineers). The social system members can often better support the interventions when they feel to be integrated, and the change process starts to be more self-sustained. It also becomes clearer who is burdened and how: this is of special interest above all in cases when so-called “index client(s)” appear(s) to be the last stable unit of an affected social system. They can still have the power to call a psychotherapist in contrast to other social system members who have not the power to care for themselves anymore. We call such phenomena a “claim of psychotherapy on behalf of others.” Multi-person conversations allow those who are protected and not apparently at the center of the psychotherapeutic action to be involved in psychotherapy anyway. Hence, the first step is to explore who should be invited (Table 4). In the IFST, we use social network diagnostics (Hunger et al., 2019; Braus et al., 2022) and genogram work (Petry and McGoldrick, 2013) to better understand the composition of affected social systems and their current as well as transgenerational social relationships.

**Table 4:** Inviting social system members into therapy (Schweitzer et al., 2020).

**Criteria for identifying significant social system members**
1. Client(s) and significant other(s) who suffer from social anxiety in a friendly and empathic way (e.g., parents, partners, friends, colleagues, and superiors)
2. Client(s) and significant other(s) who (un)consciously co-chronify the social anxiety by protection and caring (e.g., “I’ll do it for you if you are too anxious”).
3. Client(s) and significant other(s) with whom the client(s) have “unfinished business” (e.g., often, but not always: parents whose delegation was not taken by their children, or children who have not get what they perceive to have deserved from their parents)
4. Client(s) and significant other(s) who are important for stabilizing already achieved steps of change (e.g., innovative social relationships stimulated by “social drifts” from, e.g., anxiety to support social networks)

### Reflecting team

In order to give transparency to therapeutic processes and empower clients as autonomous entities, *reflective teams* can be installed in each ISFT session. The epistemological background refers to radical constructivism and the assumption that there are as many truth(s) as persons participating in the therapy, including therapists (Andersen, 1991). The forming of a meaningful difference then requires questions that have not been addressed by the clients nor the therapists. Reflecting teams strive to consider *what* constitutes the clients’ (dys)functional communication and interaction in the sense of first-order cybernetics, and to provide a positive connotation of *how* to communicate and interact with each other in the sense of second-order cybernetics.

**Figure 2:**
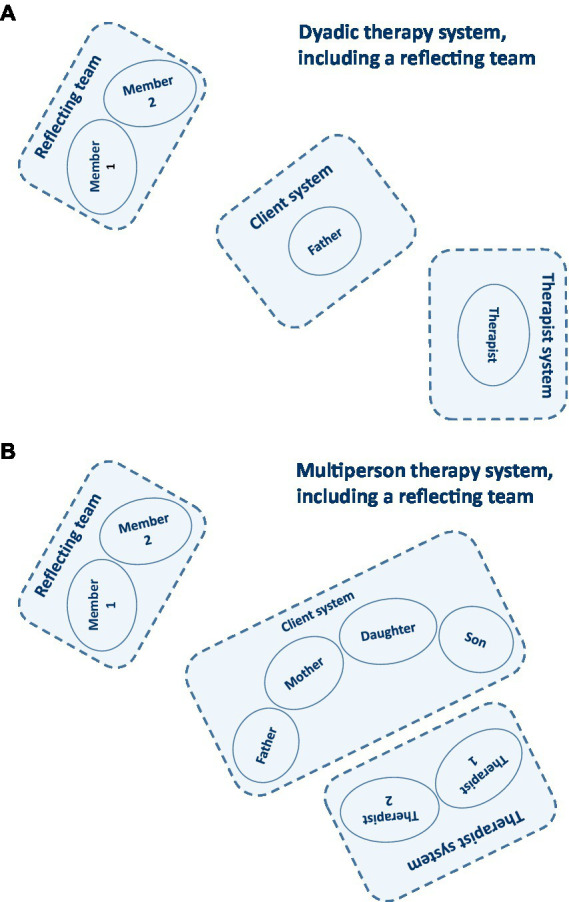
Therapy systems: **(A)** Dyadic therapy system, including a reflecting team; **(B)** Multi-person therapy system, including a reflecting team (Hunger, 2021).

In tradition to the Milan approach (Selvini Palazzoli et al., 1975), we install reflecting teams directly in the therapy room. As a subsystem observing the therapy process, the position of the reflecting team is characterized by distance while being turned towards the clients and therapists at the same time (Figure 2). The reflecting team listens attentively and formulates questions, first inwardly and later verbalized, on how the symptomatology can be explained alternatively. After a certain period of time, and in consultation with the clients, the therapist asks the reflecting team for its perceptions, including ideas and questions to what they have perceived so far. The reflecting team members talk to each other but neither to the clients nor the therapist. They talk about their perceptions (“I perceive...!”), not about truths. Likewise, they ask themselves questions that they assume to contribute to a meaningful difference in the clients’ communication and interaction patterns (“...and I wonder...?”). Subsequently, the therapist binds back to the clients and, in turn, asks for their ideas and questions in response to what they have perceived on the part of the reflecting team. The clients are thus invited *to co-create a conversation about the reflecting team’s conversation about the therapy system’s conversation*. A self-referential and self-organizing dialogue (autopoiesis) emerges about *what* serves to control the clients’ self- and social system-preservation (first-order cybernetics) and *how* this control can undergo change (second-order cybernetics). The polyphony as perceived by the clients as well as therapists, and the reflecting team’s example that this polyphony can be heard and benevolently negotiated, is considered a central mechanism of change in systemic therapy (Andersen, 1991).

### Initial phase: Joining, social networks and case construction

We perceive clients especially at the beginning of therapy, and despite their apparent problem orientation, not at the low point of their crisis. If that were the case, they would not pick up the phone to call a complete stranger but rather try to avoid any contact with people unknown to them! Clients who enter psychotherapy have usually gone through a longer decision-making process and have certain ideas of what they hope to achieve, often with less informed expectations of what awaits them (Prior, 2010).

#### Initial phone contact and initial face-to-face conversation

We try to meet as early as possible with the clients’ motivation, their pronounced suggestibility, and openness for new information as well as influences. The *initial phone contact* (approx. 20 min) serves as a first encounter between clients and therapists, and the radical constructivist and solution-oriented stance of the IFST. The first aim is to form an idea considering the symptom context. We ask the clients for a heading for their concern: e.g., “Finish the son’s apprenticeship—even if it costs our family life!” The clients are invited to sketch their therapy goal which we support with respect to a positive goal formulation: e.g., “You would be happy to see your son with a professional degree that fits well to his competencies and inner wishes, and that you both stay alive in this family process?” When therapists experience the clients’ concern as appropriate in terms of the proposed therapy, and clients agree to this, an appointment is made. The second part of this initial phone call serves the enculturation into the initial face-to-face conversation along three main topics. We ask clients if they would like to know about our interests in the first therapy session, and this question is almost always answered with a “Yes, I’d love to.” (1) We reaffirm our interest in possible solution scenarios, introducing the miracle question: “The first thing I, as a therapist, will be particularly interested in is your goals. It is important to me that we, together, develop a clear picture of where you want to be at the end of our shared time. If we assume it goes optimally and you finally say goodbye with the words ‘I am now where I wanted to be!’, I would be interested in: ‘Where are you then? How will you feel about yourself and others? What will you do differently?’ I’ll bring a lot of questions like this to our initial face-to-face conversation because I want to make sure we are pulling in the same direction.” (2) We also are interested in problem-solving strategies tried so far: “I’ll ask you in our first face-to-face conversation: ‘What have you already tried to approach your goal?”. There will certainly be some action that has made the problem smaller, and some action that has tended to make it worse. I’m interested in both: the successful, because perhaps we can do that more, and the unsuccessful, because this can save us from going in a wrong direction. Does that make sense to you?” (3) We finally point to the recognition of possible changes from now on till the first face-to-face encounter: “I finally will be interested in the good things that may have happened between our contact today till we meet face-to-face. Research has shown that over 70% of the clients who book a therapy appointment experience an improvement between these two events. This can be a small improvement as well as a very significant one—all the way to the rare case where therapy is no longer needed at all. So, I would ask you to simply pay attention to possible changes.” The clients receive these questions together with the appointment confirmation by post. If multiple members of an affected social system are involved, the initial phone contact is conducted with each system member (Prior, 2010). The *initial face-to-face conversation* follows the choreography of the initial phone contact.

#### Social network diagnostics and genogram

Another meaningful part of the initial phase is to gain a better understanding of the structure and characteristics of the affected social system. We use genogram interviews (Petry and McGoldrick, 2013) to facilitate a pronounced transgenerational system perspective of the social anxiety. Genogram work according to the ISFT includes the identification and demarcation of social anxiety in the family and its history, social trigger constellations for social anxiety and social interaction cycles for its alteration, upcoming developmental task not approached by the family but indicated by the social anxiety, solution scenarios and the motivation to change. Reflecting teams can be used at any time to broaden the perspective of clients and therapists (Table 5).

**Table 5:** Genogram interview in the context of social anxiety (Schweitzer et al., 2020).

**Process of a genogram interview**
**Data collection** “Who-is-Who in the family?” We ask for parents, siblings, (former) partners, children, grandparents, and other important relatives. We also pay special attention to excluded persons such as children that are not born or given up for adoption. **Reflecting team I** “Are the social system members named comprehensively, or are significant others missing – and if so, which ones? Is the number of social system members mentioned appropriate, or too large – and if so, which persons, or group of persons, respectively, seems most interesting for the start of the genogram work?” We use the reflecting team in the initial phase as early as possible in the intervention process to give it the chance to hypothesize as unimpressed as possible by what the client(s) otherwise may have already told us in a later stage of the intervention process. **Offerings for identification and demarcation** “Were there (social) anxiety, or other mental health problems, in your family? With whom and in which contexts?, How was this dealt with?, How has that affected you?” **Trigger constellations** “When did the social anxiety appear for the first time?, How was your family structure at that time: who was close by, who was far away?, Who noticed you, and who not?, Did anything special change in your family or way of life at that time?” We also ask for social system stress, e.g., family dysfunction, illness, poverty, migration. **Reflecting team II** “What are the family members trying to prevent, or enable?, What would have to happen to make some of the family members more present (motivation to a place more in the foreground) or hidden (motivation to a place more in the background)?, How do they respond to each other, and what may introduce a significant difference in how they respond to each other?” We use the reflecting team in the mid-phase of the intervention process to give it the chance to hypothesize as unimpressed as possible by what the client(s) otherwise may have already told us in a later stage of the intervention process. **Interaction cycles** “Who responded to the fears and how?, How did you, and others, responded to these reactions?, What was tried to alleviate the fears, and by whom?” (problem solving within 1^st^-order cybernetics) **Upcoming development tasks** “What family stories deal with loss, separation, insecurity?, Who has (failed to) provide security?, Who would tell these stories in which version?, What is next to experience?” We also ask about social system stress, if necessary. **Future meets presence** “Suppose things are going well, you wake up one day and the symptoms are gone: how are your family relationships, and what has changed?, What is a first step from this solution scenario, looking at the problematic present, to successfully attain this future present?” **Motivation to change** “What (un)wanted family changes stimulate reduction in social anxiety?, What would get better, worse, or stay the same?, Who gives you power to change, and who withdraws that power?” **Reflecting team III** *Coherence*: “How coherent does the intervention process and the (solution) picture of the genogram appear?” *Forgotten family members and/or relationships*: “What was possibly mentioned at an earlier point in time, however, then no longer addressed, but seems to be important?” We use the reflecting team in the final phase of the intervention process to give it the chance to hypothesize as conclusive as possible by what the client(s) otherwise may have missed in their conversation process. **Termination** Final reconnection from the reflecting team to clients; resume of the intervention process and take-home-messages by clients and therapists; ending of the therapy session.

**Figure 3:**
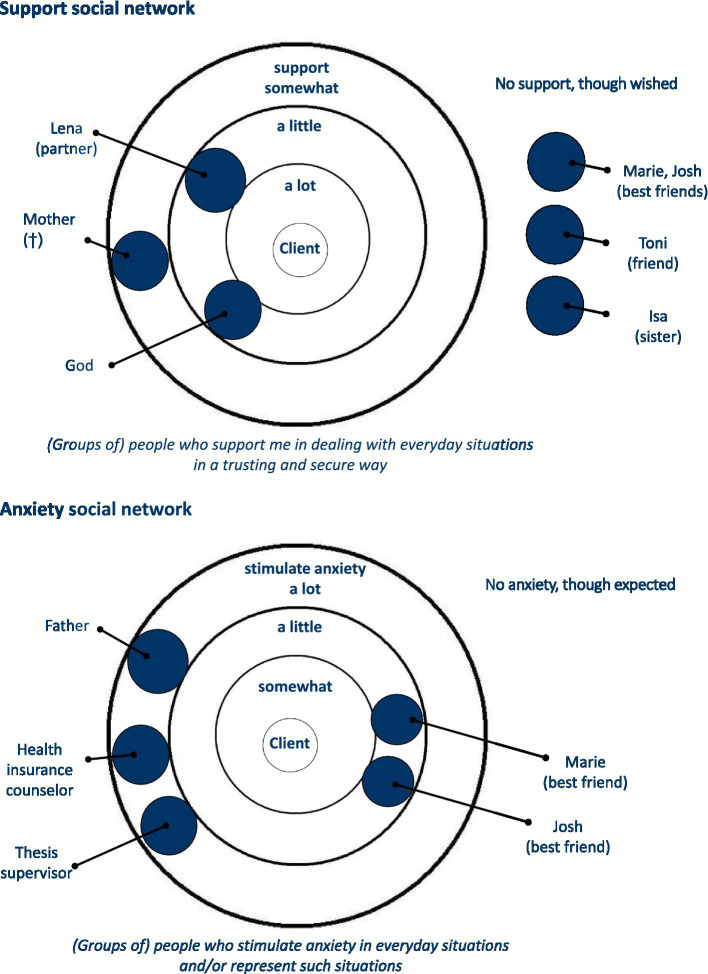
Support and anxiety social network (Schweitzer et al., 2020; Hunger, 2021).

Genograms, however, are limited to biological and legal relationships. They do not well include significant others, e.g., friends, neighbors, colleagues, and co-workers. We thus developed the Social Network Diagnostics (Hunger et al., 2019) to better understand the structure of the affected social system including all important social system members, while keeping in mind that an appropriate number (quantity) and prosociality (quality) of social relations characterize well-integrated social networks (Eaker et al., 2007). The Social Network Diagnostics uses a semi-structured interview to assess the social system’s structure based on three concentrically arranged circles. We distinguish between resource-specific and disorder-specific social networks (Figure 3). The affected social system (e.g., “I” or “my family”) is positioned at the center of the circle structure. Resource-specific social networks, e.g., support social networks, ask for (groups of) persons who provide support for dealing with everyday situations in a trusting and secure way. The client(s) place wooden stones in the first, second or third circle representing the (groups of) persons who support him/her/them a lot/not so much, but also a little/somewhat. (Groups of) persons who do not give support at all, although wished by the client(s), find a place around the circles. Structural aspects such as the network size, demography, kind and duration of the relationship, and frequency of contact as well as functional aspects, such as positive social support, social negativity, and system experience are asked about for all (groups of) persons (Table 6). The same procedure is used in disorder-specific networks, performing an inverted arrangement of the concentric circles, e.g., anxiety social networks (Figure 3).

**Table 6:** Structural and functional aspects of social networks (Hunger et al., 2019).

**Categories**		**Examples**
**Structural aspects**	**Size**	“Who belongs and who does not?”
	**Demography**	“How old is [person]?,” “What gender is [person]?”
	**Kind of relationship**	“What is your relationship with [person]?”
	**Duration of relationship**	“How long have you known [person]?”
	**Frequency of contact**	“How often do you see and/or talk to [person]?”
**Functional aspects**	**Positive social support**	“How much do you experience that [person] cares about you?” “How reliable can you turn to [person] when you have a problem?” “How strong do you feel that [person] supports you?” “How much do you feel understood by [person]?” “How intense does [person] motivate you to tackle things concretely?”
	**Social negativity**	“How much do you feel criticized by [person]?” “How strongly does [person] get on your nerves?” “How often do you argue with [person]?” “How much do you feel overwhelmed by [person]?” “How intense does [person] prevent you from doing things concretely?”
	**System experience**	“How much do you experience that you and [person] belong to each other?” “How reliable do you experience that you can be in touch with [person] about your needs?” “How much do you experience yourself in harmony with [person], meaning that you are good at staying in touch even when things do not go harmoniously?”

#### Shared case construction and therapy planning

The initial phase ends with a *circular focus formulation* which is at the heart of systemic therapy. The circular focus formulation grounds in as precise a description as possible of how the social anxiety is linked with the disturbed communication and interaction patterns. It should well explain the emergence, maintenance and possibilities for change with respect to the social anxiety within the affected social system. The aim is to provide an approach how to alter the symptomatology while stabilizing previously identified resource-oriented relationships and modify problem-oriented communication and interaction patterns.

An example may serve as an illustration: A client experiences how the refrigerator is getting emptier, and he suspects that he will soon have to go shopping. He asks his partner to do the shopping. The partner reacts annoyed. The client’s body posture becomes more tense with each refusal. The partner shows increasing distance with each new request. Finally, it comes to a dispute, and the partner strongly annoyed leaves the room while the client strongly annoyed stays at home. The opportunity for a joint solution seems to have been missed. According to Rotthaus (2015), the following questions now can guide the process of working out a circular focus formulation:

1. “What is the function of the social anxiety with respect to (the prevention of) the further development of the affected social system? What message does it send to which system member?”
2. “What role do (invisible) loyalties play? Which family, individual and context-related communication and interaction patterns (e.g., attachment (in)security, devaluation, and exclusion) are part of the background of the social anxiety?”
3. “How have similar challenges been (not) successfully dealt with?”
4. “Assuming that the social anxiety has become redundant: how will the client(s) live, love, and work?”

*Reframings* serve the positive reinterpretation within the shared case construction. They enable reversals into the opposite, as well as role reversals when therapists occupy the position of the “sceptics,” valuing symptoms, and questioning change. Complementarily, they allow clients to take a more active position as “convincers that the solution is possible.” In a variation of Schwing and Fryszer (2006), the formulation of a good reframing follows a narrow sequence of five steps:

1. “What is it exactly that is disturbing you? Please, describe the socially anxious behavior and experience specifically.”
2. “In what contexts does socially anxious behavior and experience fit well, i.e., appears appropriate? In what situations was, and still is it, meaningful?”
3. “What skills become evident in the context of the social anxiety? What have/can you learn from it?”
4. “What good intention do you attribute to the social anxiety? What do you, and others, (un)consciously want to achieve (with the social anxiety)?”
5. “What alternative behavior and experience appear in the context of meaning-making and maintaining the good intentions of the social anxiety, thus taking advantage of it? How can you design the solution in a way that it still contains the positive elements of the problem?”

A circular focus formulation, including a reframing, can explain the function of the social anxiety in the above described couple: The social anxiety of one partner keeps hold of the social system by involving the other partner’s responsibility. The less one partner goes into contact with the outside world, the more the other partner takes over. Transgenerational (invisible) loyalties may operate in the background and motivate one partner more than the other to keep a low profile and not dare risk the encounter with others. The family history showed that avoiding attention was essential to survive in war times. This was truer for a Jewish family like the partner’s family of origin who indicated social anxiety symptoms. Now, however, the war is over, and the parents’ education continues to have an effect on this partner and prevents the further development of the couple as the affected social system. If the social anxiety becomes redundant, the previously social anxious partner explains that he would intensively like to enjoy the new freedom. This, in turn, provokes anxiety in the other partner, who up to now has represented the couple to the outside world, and was happy not take too great a leap. What is needed is not simply a solution to the social anxiety symptoms, but rather a shared construction of what life could be like without the previously shared social anxiety. This would include altered and healthier communication, and interaction patterns.

### Mid-phase: Experimentation

In this stage of therapy, it is important to try out potential *opportunities for change (“experiments”)*. The therapeutic stance is of high importance, especially when outreach interventions and innovative solution scenarios are performed in the everyday environment of the affected social system. Relevant to action is an *optimism for change without pressure to change*, framed by caring humour while playing with symptoms and having fun with friendly absurdity and unusual experiments. Core interventions include the working with circular questioning and hypotheses (Penn, 1982; Cecchin, 1987), symptom prescription in accordance with the Milan approach (Selvini Palazzoli et al., 1975), and choral speaking (Schweitzer et al., 2020; Hilzinger et al., 2021).

#### Systemic questioning and hypothesizing

Systemic questioning and hypothesizing, especially addressing circular phenomena, allow a better understanding of the communicative and interactive vicious circles of an affected social system. Circular questions address dyadic interactions, e.g., when a therapist (A) asks how one person (B) thinks about another person (C). Similarly, triadic interactions can be considered when the therapist (A) asks how a person (B) experiences the interaction of two or more other persons (C, D, etc.). This way of asking may appear strange at first, but what is asked is part of our daily life: we do not only react to what others do, but rather to what we think others think about us (expectation–expectation). When all social system members are involved in the therapy session and experience what one system member thinks about the other, then everyone learns something new (Simon and Rech-Simon, 1998). The goal is to make clear that every communication embodies a content and relationship aspect and that in disruptive events it is usually less about the content but more about the underlying unmet needs within the social system. Well-informed alternative ways of communication consequently can be developed and tested for a more appropriate conflict resolution. There are various modes of systemic questioning that serve this purpose, including solution/wonder questions, contextualizations, operationalizations, aggravations, optionalizations, historical questions, and scalings (Table 7).

**Table 7:** Modes of systemic questioning (Hunger, 2021).

**Modes**	**Examples**
**Solution questions, wonder questions** *Goal actualization*	“Suppose you solve the problem, e.g., the (social) anxiety, who notices it first? What is noticed? What are special features of the situation without the (social) anxiety?”
**Contextualizations** *Liquefaction of traits into behaviors*	“How do you manage moments with and without (social) anxiety? How do significant others manage such situations? How do others manage to show themselves straight and/or in need for help? Do they all and always show themselves like this, or is it in certain contexts and at certain times?”
**Operationalizations** *Explanatory models*	“How does each social system member explain other’s thinking, feeling, and behavior?”
**Aggravations** *Problem intensification*	“What makes a good contribution to a faster and/or stronger escalation of the situation?”
**Optionalizations** *Alternative constructions of reality*	“Suppose someone decides to resist the invitations of others, and to stop presenting him- or herself as a conspicuous person, e.g., with (social) anxiety: How will this change challenge the established relationships? Who welcomes this change? What is missed? For whom does this change seem less acceptable?”
**Historical questions** *History of the symptomatology*	“When do the individual social system members think they first noticed the client(s) symptomatic behavior? Why not sooner, or later in time? When do others think that a certain person within the social system feels out of sorts?”
**Scaling**	“How do the client(s) rate the possibility to live a life without (social) anxiety? How real do the affected social system members rate the possibility of the intervention process failing?”

#### Symptom prescription: “If you have a symptom—Use it!”

The Milan approach (Selvini Palazzoli et al., 1975) was in close exchange with the Heidelberg School (Stierlin, 1976). Epistemologically, *symptoms are understood as an independent element* indicating the quality of an affected social systems, functioning. To introduce a difference in the therapeutic setting that makes a meaningful difference (Bateson, 2000), clients are invited to perform something unexpected, i.e., to intentionally (not) perform the symptom components (e.g., physiological, behavioral, cognitive, and emotional). This is associated with symptom reduction (e.g., less shaking and/or blushing in social anxiety disorders) or symptom escalation (e.g., strong shaking and/or blushing). The aim is no longer at the solution of the symptoms as this has already been tried many times in vain. Contrariwise, symptom prescriptions proved to be particularly helpful when change had failed, or too early changes bore the risk to threaten the survival of the affected social system. For example, symptoms of social anxiety may have been reduced from 6 h to 3 h the day before the performance of a speech; if, however, attractive alternative goals are missing, responding to the question with whom and what one will spent the 3 h of gained free time, suicidal tendencies can arise to fill the increasing emptiness.

The symptom components are usually prescribed in isolation. Positive symptoms (e.g., “Do [show, think, feel] what you already do [show, think, feel]”) can be addressed (e.g., “Try to sweat as strong as possible, and even stronger, before meeting strangers!”; “Shake so much that you tip over your glass of wine at the garden party and submerge your mother-in-law’s dinner!”). Likewise, negative symptoms (e.g., “Do not do [show, think, feel] what you do not do [show, think, feel]”) can be addressed (e.g., “Do not tell anyone that your career aspiration actually is totally different to your father’s ideas!”; “Be sure to stay at the bar next to the dance floor in the discotheque and by no means dance with your friends, even if your favourite song is playing!”).

“Such ritualized prescriptions of communication and interaction patterns create an exacerbation of the situation in a humorous way. They make clear what is going on, respect it as meaningful, and create a certain pressure not to keep up this nonsense” (Schlippe and Schweitzer, 2016, p. 334).

**Figure 4:**
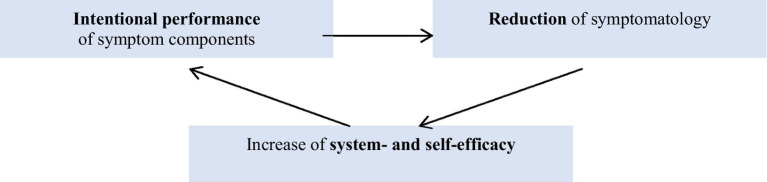
Rationale of symptom prescription (Hunger, 2021).

The goal of symptom prescription is to deliberately perform selected symptom components. The affected social system decides when certain symptoms are allowed to come on stage. This way of dealing with symptoms causes a reduction in the symptomatology and increases the affected social systems’ experience of system- and self-efficacy (Figure 4).

The framing of the symptom prescription can be performed as directive as in the 1970th Milan approach, including a confronting closing commentary (Selvini Palazzoli et al., 1975). In the ISFT, we have adapted the Milan approach based on our experience in nowadays systemic therapy. Creating an arc of tension, options for scepticism on the one hand and the commitment of all social system members on the other hand are of particular importance to increase the success of the intervention (Table 8). In addition, we have developed a three-step approach by prescribing *problems*, *solutions*, and *avoidances*. The actual performance of the symptom prescription (*in vivo*) is as valid as its hypothetical enactment in the context of system- and self-reflection procedures (*in sensu*). It is important that the situation to be addressed, and the behavior to be proved, are planned together with the social system members and in accordance with their goals, resources, and the social contexts that arise curiosity (Table 9).

**Table 8:** Closing commentary introducing a symptom prescription (Schweitzer et al., 2020).

**Process**	**Example**
**Summary**	“We [therapists] have been talking to each other, and our impression is that you are a very committed family: the fact that you have managed to come here as a four-person family and engage with each other in the hope of getting better, that’s truly remarkable.”
**Invitation to participate**	“We came up with an idea and we invite you to try something out.” *Important: Control remains with the affected social system; it is allowed to deny the invitation to experimentation!*
**Creation of an arc of tension**	“Of course, trying something new is always risky, too, so it’s good for you to think carefully about whether you are ready to take that risk or not.”
**Enabling scepticism**	“The risk is that you can be faced with a change of your situation, for better or for worse! You may see advantages and disadvantages in that change, and perhaps you have already expressed them.”
**Commitment of all social system members**	“We cannot start the experiment until everyone is really ready and feels confident enough to engage in this experiment.” *Important: Ask for a true commitment and wait until each social system member is ready! Possibly let another (few) therapy hours pass before addressing the symptom prescription again.*
**Formulation of the symptom prescription**	

**Table 9:** Three-step approach of symptom prescription (Schweitzer et al., 2020).

**Steps**	**Task, system- and self-reflection**
Problem prescription	Task: e.g., Tuesday, 7–7.30 pm: Preparing spaghetti with tomato sauce, with all the *problem* symptoms at hand, including the partner. One partner cooks, shakes, sweats, oversalts the pasta, spills the tomato sauce, and trashes the kitchen. The other partner watches TV with friends and calls into the kitchen in the meantime: “Honey, is everything okay? It smells so burnt!” System- and self-reflexion: Both clients reported a very stressful week. The symptom prescription could not be implemented.
Solution prescription	Task: e.g., Tuesday, 7–7.30 pm: Preparing spaghetti with tomato sauce, with all the *solution* symptoms at hand, including the partner. One partner cooks, having all utensils well-arranged at the beginning of the scene, and listens to his favorite classics while first preparing the pasta and then the tomato sauce, well-seasoned. The other partner watches TV and comes into the kitchen in the meantime, looking over the cook’s shoulder, smelling with pleasure the aroma rising from the pots and says with a kiss: “Honey, that smells so really delicious, I’m truly looking forward to our meal!” System- and self-reflexion: Both clients reported a very stressful week. The symptom prescription could not be implemented.
Avoidance prescription	Task: e.g., Tuesday, 7–7.30 pm: Preparing spaghetti with tomato sauce, with performing an avoidance situation by arranging the meals on the table and subsequently leaving the house without a word and any reaction of others. System- and self-reflexion: All clients reported a less stressful week. The symptom prescription was performed. When the door was about to close on leaving the house, the cooking partner felt the urge to know what his meal tasted like after all. However, the door closed. The cooking partner had forgotten the key, and ringing the bell was not possible in this moment. In his first thought, he realized: “I want to be evaluated, but preferably positive, not negative!” The second thought showed: “For positive evaluation I need social contacts!” The third thought included: “If I avoid evaluating situations, I exclude myself. That has a negative impact, above all on myself, because I also exclude possible compliments and the joy of being together with my friends! In addition, it works also most negatively for my friends who likewise experience to be excluded from my life at the time when I am outside and not part of the company at table!”

#### Choral speaking: Turning self-doubt into rhythm and music

Incisive negative beliefs represent mental models which have the power to entrap a person into a so-called problem trance. A problem trance is usually referred to as a trance state that arises when a client mentally enters a subject of high emotionality (Schlippe and Schweitzer, 2016). Such beliefs can be recognized by the following phenomena: (1) sentences in which one blames or accuses oneself, (2) sentences that are emotionally charged but not necessarily explicit, and (3) sentences with which one becomes discouraged, resigned, or fearful. With the choral speaking method (Schweitzer-Rothers, 2006), these sentences can be externalized and beliefs expressed in the sentences can be questioned. The stressful emotions are set in motion, become more and more flexible and distanced from the clients. The goal is to broaden both the clients’ thoughts and bodily sensations which co-carry these feelings, till new solution-oriented ideas emerge in kind of a “solution trance.” Choral speaking is easier the more people are present. The smaller the therapy system, the more the therapist must serve as both conductor and choir.

In line with the ISFT manual, the supervisor, six clients and their therapists met once during a therapy in a group session which lasted 3 h. The first part consisted of getting to know each other, with a special focus on the different goals that clients wanted to achieve within the therapy. The second part was essentially the choral speaking method, where clients’ belief systems are sung by the group until the clients begin to show altered reactions. (1) Clients write down their individual answers to the following questions on a flip chart: “What scares me?,” “Who is to blame?,” “What cannot I change?,” and “What would I have to do to make it worse?” (problem-trance). Likewise, the following questions are noted: “Where do I feel safe and in good hands?,” “With whom do I experience such moments?,” “What can I change?,” and “What can I do to make it more like I want it to be?” (solution-trance). (2) This is followed by an exchange in small groups of two or three clients and/or therapists. (3) Together with all clients and therapists, the sentences which have the strongest power to knock someone down as well as the sentences associated with intense positive emotions are identified. (4) Two large choruses are formed. The “problem choir” intones the most concise problem-trance sentences. The “solution choir” intones the most concise solution-trance sentences. The choirs each sing for one client. The client stands in front of the choir, with the supervisor as the conductor, and lets the sung sentences have an effect on him or her. (5) Problem-trance sentences are sung aloud or quiet, fast or slow, at least 10 times, with a short break between each repetition, until the client’s reaction changes. This can be anger at oneself (e.g., “Why do I torture myself so much!”), differentiating ideas (e.g., “That’s not always true!”), re-evaluations (e.g., “The second row gives much more freedom for self-care than the stressful positon of a front woman!”), or new posture ideas (e.g., laughter, or the song “The Bare Necessities” in remembrance of the Jungle Book). The new impulse is introduced into the chorus as a new phrase. The majority of the singers continue to sing the old phrase while a minority alternates singing the new phrase. In a singing contest, the conflicting movements compete against each other. In the listening client, new, third, fourth, fifth, etc. movements are often perceived. These reactions are integrated into the concert by their performance as new voices increasingly differentiating sub-systems of the choir. The process ends when the listening client feels the increase of (more) power and energy, or at least peace and calmness. (6) Solution-trance sentences are sung shorter. Their listening represents a ceremony. If they are botched, they lose their effect. When the listening client has recorded an inner soundtrack of the choral speaking, the choir ends. (7) A debriefing closes the choral speaking methods.

### Final phase: Stabilization, prolapse prevention, and evaluation

At the end of the ISFT, we and the client(s) reflect on the path we have travelled together in therapy. We congratulate the members of the affected social system on their progress and success. The focus is on the *stabilization* of the changes achieved. The goal is to make experiences from the therapy helpful for dealing with possible and expected “re-invitations” to future social anxieties in the sense of *prolapse prevention*. This includes exploring negative consequences of missing future *rounds of honor in the old pattern*: “What will be missing and become more stressful or conflictual if others experience you as less anxious in the future?.” This is often followed by a collection of good reasons for future prolapses: “What are benefits you associate with staying more or less socially anxious?”

We use *timeline work* (Suddaby and Landau, 1998) and allow for a glance into the future: “How and with whom do you (not) live, love and work in 3, 6, or 12 months?, What important things have (not) happened when and with whom?, Which challenges have (not) been mastered with whom and how?, Which challenges still have to be (not) overcome with whom and how?” *Balances* clarify (1) what has been changed, and which social system members contributed, (2) which change steps have (not yet) been performed, and (3) how life can continue even with the unchanged. The goal is that all social system members applaud themselves and the others for what has been achieved and give their blessing to be able to find peace with what currently could (not) be changed or will (not) be changed in the future. A shared *farewell ritual* serves as a transition to a life without therapy. A *comeback* is also possible. Even if we make a clear cut and do not extend therapies beyond what is refunded by the national health insurance, we remain open to meeting again after an appropriate therapy break based on success. The length of this break is negotiated with the affected social system. The comeback neither depends on the social system members being in a current crisis. It rather becomes possible with the presentation of a new concern which could not be well addressed in former therapy hours.

## Pilot randomized controlled trial

Following the studies of Willutzki et al. (2004), Rakowska (2011), and Leichsenring et al. (2009), we aimed to investigate the IFST for social anxiety disorder (SAD) considering its feasibility and trends of change in psychological, social system, and global functioning. This section will summarize the findings published in the original pilot RCT (Hunger et al., 2016a, 2020). The interested reader can find a detailed review of studies and evidence of psychotherapies for the treatment of SAD elsewhere (Hilzinger et al., 2016).

### Design

We used the well-established Cognitive Behavioral Therapy (CBT) as a comparator (Clark and Wells, 1995; Stangier et al., 2016). Previous studies of systemic therapy for SAD have focused on individual therapies (Willutzki et al., 2004; Rakowska, 2011). The study by Leichsenring et al. (2009), the probably largest psychotherapy study on SAD, exclusively compared CBT with Psychodynamic Psychotherapy.

We conducted a prospective multicenter, assessor-blind randomized controlled trial (RCT; CBT: Center for Psychological Psychotherapy; ISFT: Institute of Medical Psychology; Ethics Committee of the Medical Faculty of Heidelberg University: S-190/2014; registered with the U.S. National Library of Medicine, ClinicalTrials.gov: #NCT02360033).

### Methods

#### Sample size calculation and randomization

According to Cocks and Torgerson (2013), we aimed to recruit a minimum of 32 patients for a powered two-arm pilot study. Considering a possible drop-out rate of 25%, we decided not to stop recruitment until 38 patients were enrolled and allocated. An independent allocator team performed block randomization to CBT or ISFT, and subsequently randomized patients to therapists (Efird, 2011). They then sent assignment information to the study director (CHS), who forwarded them to the staff members (Hunger et al., 2020).

#### Patient, social system members, and therapists

We screened 252 interested persons, and of these, 38 *patients* were allocated to CBT and ISFT, respectively (CBT: 20 patients; ISFT: 18 patients). The patient flow can be found elsewhere (Hunger et al., 2020). Patients were almost equally men and women in their 30s with similar education levels, mainly married or living with a partner. *Social system members* were mainly married or living with a partner, well-educated and employed spouses or partners, parents, (best)friends, children, or siblings. *Therapists* were mostly educated females in their 30s, and the majority was married or living with a partner. Study arms were well-balanced with respect to patients’ data at baseline.

#### Therapist training, adherence, and allegiance

No therapist had practiced the CBT or ISFT manual before the trial started, so therapists all participated in three 3-day CBT or ISFT *manual trainings*. Subsequently, every therapist performed a training phase including the treatment of two patients. Experts in CBT and ISFT provided supervision every fourth therapy hour. Over the course of the study, CBT therapists’ global *adherence* showed smaller deviations (CTAS-SP: *M* = 2.18; *SD* = 0.29; 0 = no adherence, 3 = very good adherence; Consbruch et al., 2008). ST therapists’ global adherence was frequently demonstrated (STAS: *M* = 2.51; *SD* = 0.66; 0 = not at all, 3 = very often; Hilzinger et al., 2016; Hunger et al., 2020). In accordance with Borkovec and Nau (1972), we asked for the therapists’ *allegiance* to either CBT or ISFT (i.e., CBT: “How enthusiastic are you about CBT?”; ISFT: “How enthusiastic are you about ISFT?”; 1 = not at all, 5 = very much). Therapists’ allegiance did not differ between study arms (CBT: *M* = 3.95, *SD* = 0.59; ISFT: *M* = 4.10, *SD* = 0.38; *t*(31) = 0.868, *p* = 0.392).

#### Comparator intervention

The CBT manual (Clark and Wells, 1995; Stangier et al., 2016) works with the individual patient aiming at (re-) establishing a realistic self-perception in five therapy phases: (a) generation of an idiosyncratic version of the disorder and identification of safety behaviors; (b) manipulation of self-focused attention and safety behaviors, including role play and video feedback; (c) training in attentional redeployment and reduction in safety behaviors through behavioral experiments (expositions), cognitive restructuring and changing of dysfunctional convictions; (d) relapse prevention; and (e) refreshment and consolidation. Sessions were performed weekly and in the phase of relapse prevention every 2–3 weeks. Therapy sessions were mainly 50 min long, but with allowances to extend up to six sessions to a maximum of 100 min to facilitate behavioral experiments.

### Results

We will summarize the results of our pilot RCT in an overview, concentrating on the estimation of effects based on Cohen’s *d* for dimensional between- and within-group effects, and Cohen’s *h* for categorical between-group differences (Cohen, 1988). A detailed description of all instruments and results, including all test statistics and calculations, can be found in the original publication of the pilot RCT (Hunger et al., 2020).

Within-group, simple-effect intention-to-treat analyses of the *patients’ ratings on the primary outcome* showed a significant reduction in social anxiety (Liebowitz Social Anxiety Scale, LSAS-SR; Rytwinski et al., 2009), with large effects seen in both conditions from baseline to end of therapy (CBT: *d* = 1.04; ISFT: *d* = 1.67). The intention-to-treat mixed-design ANOVA comparing CBT and ISFT showed a significant large effect to the advantage of ISFT (*d* = 0.81). Per-protocol analyses supported these results.

Considering the *secondary outcomes, blind diagnosticians* use the Structured Clinical Interview (SCID; Wittchen et al., 1997; First et al., 2016) and rated seven CBT patients (46.7%) and 14 ISFT patients (77.8%) as no longer demonstrating clinically relevant SAD symptoms at the end of therapy (*χ^2^*(1) = 3.422, *p* = 0.083; IRR at 94%, range: 91–100%). Within-group, simple-effect intention-to-treat analyses showed their ratings pointing to significant improvement in global functioning (GAF; Aas, 2010) in both conditions with large effects (CBT: *d* = 0.92; ISFT: *d* = 1.50; IRR at 94%, range: 82–100%). The intention-to-treat mixed-design ANOVA showed a significant medium effect to the advantage of ISFT (*d* = 0.76).

Within-group, simple-effect intention-to-treat analyses of the *patients’ ratings on the secondary outcomes* showed a significant improvement in psychological functioning on the Beck Depression Inventory (BDI-II; Kühner et al., 2007) in both conditions (CBT: *d* = 0.50; ISFT: *d* = 1.71). The intention-to-treat mixed-design ANOVA showed a significant medium effect to the advantage of ISFT (*d* = 0.77). Significant improvement was also observed in within-group, simple-effect intention-to-treat analyses on the Global Severity Index (GSI) of the Brief Symptom Inventory (BSI; Geisheim et al., 2002) in the ISFT (*d* = 1.89), but not in the CBT. The intention-to-treat mixed-design ANOVA showed a significant medium effect to the advantage of ISFT (*d* = 0.77). Considering social system functioning, within-group, simple-effect intention-to-treat analyses of the Experience in Social Systems Questionnaire (EXIS.pers; Hunger et al., 2017) showed a significant improvement in both conditions (CBT: *d* = 0.23; ISFT: *d* = 1.06). The intention-to-treat mixed-design ANOVA was not significant.

Within-group, simple-effect intention-to-treat analysis of the *social system members’ ratings on the secondary outcomes* showed a significant reduction on the psychosocial Burden Assessment Scale (BAS; Hunger et al., 2016b) in both conditions (CBT: *d* = 0.56; ISFT: *d* = 0.59). The intention-to-treat mixed-design ANOVA was not significant. Significant improvement was also observed in within-group, simple-effect intention-to-treat analyses on the GSI in the ISFT (*d* = 0.14), but not in the CBT. Additional outcomes can be found elsewhere (Hunger et al., 2018, 2020).

Considering *clinical significance*, the level of patients’ remission (LSAS-SR) in CBT was 15%, response 55%, no change 25%, and deterioration 5%. For ISFT, the level of remission was 39% (*h*: 0.55), response 56% (*h*: 0.01), no change 1% (*h*: 0.57), and deterioration 0% (*h*: 0.45; Hunger et al., 2020).

## Discussion

We developed a manualized disorder-specific ISFT for SAD, evaluated for its feasibility in a multicenter, assessor-blind pilot RCT, and compared it to manualized and monitored CBT (Clark and Wells, 1995; Stangier et al., 2016). The discussion will concentrate on recommendations for the use of the ISFT manual in further studies, and for a confirmatory RCT to test the reported effects on psychological, social system and global functioning including both the patients and their social systems (e.g., family, couple; co-workers).

### Acceptability of the manual and the interventions

#### Manual structure

At the beginning of the ISFT project, the manual structure was designed strictly parallel to the number of hours and sequence of sessions of the CBT manual (Stangier et al., 2016), as this was the comparator. Initially, the therapy sessions followed each other closely, often weekly, and more sessions were agreed upon than proved useful and necessary. Therapists reported a feeling of “methodological pressure” from the manual: e.g., “I thought that I have to have my genogram interview ready after the second session as it is part of the initial phase. So, if I wanted to keep to the manual structure, I thought I had to hurry.” In the course of our pilot RCT, the ISFT therapists increasingly designed their own style of how to use the manual. They allowed themselves to omit manualized interventions, e.g., a third symptom prescription after two previous ones that had already been successful, when patients and therapists did not expect it to bring about further meaningful difference. At the end of the project, the ISFT dosage demonstrated a minor number of therapy hours compared to the manualized 25 h, and to the comparator in the per-protocol-analysis (CBT: *M* = 26.00 h, *SD* = 0.00, no range; ST: *M* = 22.50 h, *SD* = 2.57, range: 17–26; *t*(31) = 42.524, *p* = 0.000, *d* = 2.48). These findings support our stance toward the perception of the ISFT manual as an ideal suggestion from which there are good reasons to deviate in individual cases. As we already said in the ISFT introduction above, it seems optimal to us *to take the ISFT manual sufficiently serious, but not too serious*. This is why all interventions are interchangeable in their order, and why therapy planning in the ISFT can already start in the first therapy hour. It has not to wait till, all information have been collected at the end of the initial phase.

#### Systemic interventions

Therapists also reported that the ISFT manual had made it possible “for me to approach a lot of things more quickly.” The use of the *initial phone contact* (Prior, 2010) allowed the therapists to work solution-oriented already before an encounter with the clients. It turned out to be an enculturation into the ISFT. Clients no longer came to the therapy with the expectation of having to present as many problems as possible in order to get access to treatment (“ticket to admission”; Goldberg and Bridges, 1988). In none of the ISFT patient-therapist-dyades did we perceive the “culture clash” often described in the practice of systemic therapy. This becomes evident when patients believe that they must communicate problems while therapists strive for solutions. This phenomenon also includes social system members when present during the ISFT.

The *Social Network Diagnostics* (Hunger et al., 2019) was mentioned by the clients, diagnosticians, therapists and researchers to be very useful for the detection of social system members, e.g., partners, family members, and other important caregivers. Both diagnosticians and therapists described the conductance of the Social Network Diagnostics on par with the SCID interview and highly supportive to include significant others in the therapy process either as significant relative or friend, and/or additional client with clinical problems. Therapists also reported that the Social Network Diagnostics made it easier for them to address and negotiate changes of social relationships which is at least as important as changes of SAD symptoms detected with the SCID.

Therapists also took methodological suggestions from the ISFT manual. This was most often the idea in case of the *symptom prescription*. Due to the historical closeness of the Heidelberg School (Stierlin, 1976) to the Milan approach (Selvini Palazzoli et al., 1975), we ascribed a great importance to this classical and nowadays still innovative systemic method. The therapists particularly liked our adaption of the Milan approach into a three-step approach by prescribing problems, solutions, and avoidances either *in vivo* or in *sensu*. The current German landscape of systemic therapy appears to incorporate a pronounced solution-orientation. As a result, the symptom prescription with its directive nature is rarely and less explicitly trained. In the ISFT, solution prescriptions invited therapists and patients to make a first encounter with the symptom prescription. As a result, a curiosity arose on the part of both therapists and patients to try out problem prescriptions as well. Avoidance prescriptions were experienced as particularly tricky. They often highlighted the price patients paid to protect themselves from negative criticism, making it impossible, for example, to experience any positive feedback simultaneously.

The obligation to conduct *multi-person conversations* at least once in each therapy phase encouraged the therapists to conduct settings with more than one representative of the affected social system. Additionally, the *choral speaking* was new to therapists and patients and became one of the core interventions to stimulate meaningful change from the patients’ and therapist’ viewpoint (Hilzinger et al., 2021).

### Study procedures

#### Recruitment

A larger budget for the recruitment of *patients* is needed in future RCTs. We screened 252 individuals, and of these, 189 were heard on initial screening phone calls, each lasting about 20–30 min. SCID interviews were performed with 101 individuals lasting about 60–90 min. This costly procedure was required to finally include 38 patients in the pilot RCT. Though the drop-out rate was zero for the ISFT, it was at 25% for the CBT. The budget for recruitment for this pilot RCT was inadequate and comprised the timeliness of the study as well as the more advanced investigation of therapists’ adherence and competence which is crucial for the sophisticated interpretation of study results.

There are often difficulties reported with respect to the inclusion of *social system members* in psychotherapy research. In our pilot RCT, however, this was not the fact but rather an easy game. Based on our experiences from our pilot RCT, we recommend the early application of the Social Network Diagnostics (Hunger et al., 2019) as it allows for the identification of those social system members who appear to play an important role in the development, maintenance and change of the addressed symptoms.

It was difficult to recruit *therapists* with substantial experience in multi-person settings. Although it is seen that the work with families, couples and social networks is at the core of systemic therapy, it is evident that currently multi-person settings are hardly trained in German psychotherapy. Therefore, future studies should give a special focus in the ISFT manual training and supervision of therapies like we implemented in our pilot RCT.

#### Diagnosticians

The insufficient funding for recruitment procedures equally applies to the budget for blind diagnosticians. Currently, the hourly rate for external psychological diagnosticians is about 100€, if they are not permanently employed due to a lack of funding. We saw about 100 interested persons in 60–90 min SCID interviews. Again, the funding was insufficient and should be better supported by appropriate structural working conditions. so that diagnosticians can be hired for the study period.

#### Randomization

The randomization was appropriate and the independent allocator team worked well performing block randomization (Schulz and Grimes, 2007) using a pseudorandom number generator (www.randomization.com; McLeod, 1985). Patients, social system members, and therapists knew which study-arm they were being allocated to, though not about the specific research questions. We do not see this as a disadvantage of our pilot RCT as transparency is a fact of “real word delivery of care” (Zwarenstein et al., 2008, p. 6).

#### Control group design

CBT as an active comparator worked well in our pilot RCT. It, however, showed a 25% drop-out after the initial clinical interview. The reasons for this were the demand for a stronger integration of the partner and/or family into therapy, the experience of therapy demanding too much or the detection of another primary diagnosis compared to SAD.

Furthermore, essential characteristics of systemic therapy were abandoned in favor of comparability between the ISFT and CBT as the active comparator. Systemic therapy grounds in a collective intervention culture that meets with the affected social system in multi-person settings approximately every 3–4 weeks (Schweitzer, 2014). CBT, however, belongs to individual intervention cultures and sees mostly one single patient each week. The preconditions thus were uneven to the disadvantage of the ISFT. Future studies should investigate whether differences may appear less between different schools of psychotherapy than in the nature of the performed setting. It can be assumed that by treating an entire social system, the relapse rate of individual members with previously diagnosed mental disorders appear reduced (Morgan et al., 2013). This may be due to a better balance of interpersonal in addition to intrapersonal conflicts, the recognition and multidirectional negotiation of differences in each social system members’ need for related autonomy (Stierlin, 1976) and increased options for the evolvement of an integrated prosocial support within the affected social system (Holt-Lunstad et al., 2010).

#### Instrument and test administration

The reported instruments are validated, easy to administer and impactful measures that serve well as primary and secondary outcomes for ISFT in SAD. This is even more successful as the primary outcome of ISFT is not a symptom reduction but an improvement of social system functioning. The primary effect of ISFT, assessed with the LSAS as an instrument asking for social anxiety symptoms, was therefore measured with an instrument that is less close to the actual intent and mode of action of the ISFT. Future studies should concentrate on a broader acquisition of the social system functioning, considering its different facets.

Online data collection worked well in kind of a “Data Café,” accompanied by cakes, cookies and/or coffee, which we implemented in a comfortable room for both study arms. Study staff was always available to answer questions. Since there was no fundamental criticism against the online assessment *via* the online platform UNIPARK, we recommend online data collection in future studies for economic reasons with respect to the study management, and to ensure no missing or potential data entry errors in the assessment procedure.

### Outcome trends

The statistical results need interpretation with caution, since the nature of a pilot trial is its small sample size that is not sufficiently powered to test hypotheses of program efficacy. Our pilot trial, however, used an adequate power for a two-arm pilot RCT based on the rationale of Cocks and Torgerson (2013). The trend obtained in the LSAS as the primary outcome for psychological functioning was positive and encouraging. Results also indicated significant treatment effects on additional aspects of psychological and social system functioning to the advantage of the ISFT, including blind diagnosticians’ ratings of patients’ remission from SAD as well as their global functioning. Social system members likewise reported a reduction in their psychosocial burden, and improvement of psychological functioning. This finding fits well into the socio-psycho-biological explanatory model (Figure 1; Luhmann, 2017; Hunger et al., 2018): changes in one person are reciprocally associated with changes in the other person (“spill-over effect”; Keeton et al., 2013), pointing to mental disorders as interpersonally shared realities and the need to include all important social system members in psychotherapy to empower sustainable change (Morgan and Crane, 2010). The overall positive trends of the ISFT compared to CBT in our study bode well for a larger powered RCT.

## Conclusion

Our manualized disorder-specific new ISFT for SAD was evaluated for its feasibility in a multicenter, assessor-blind pilot RCT, compared to manualized and monitored CBT. Both the creation of the manual, its acceptability by therapists, patients, and social system members, as well as the efficacy trends calculated for the ISFT bode well for a subsequent confirmatory RCT. The pilot findings indicated integrity of the study methods and procedures, a favorable acceptance of the manual by therapists, patients, and social system members. We however suggest minor adjustments to recruitment, instruments, test administration, and a stronger emphasis on the flexibility of the ISFT manual. The promising results indicate a fully powered RCT concentrating on the social system functioning, in addition to the assessment of patients’ symptomatology, to be feasible and worth of future investment of time, effort, and funding.

## Data availability statement

The datasets presented in this article are not readily available because raw data cannot be anonymized. Requests to access the datasets should be directed to CHS, christina.hunger-schoppe@uni-wh.de.

## Ethics statement

The RCT involved human participants. It was reviewed and approved by the Ethics Committee of the Heidelberg Medical Faculty (S-190/2014). The patients provided their written informed consent to participate in this study.

## Author contributions

CHS, JS, and RH conceptualized and designed the RCT. CHS, LK, LD, RH, JM, and AS contributed substantially to the data analysis, and together with JS and HB contributed to the interpretation of the study results. JS drafted the first German version of the ISFT manual, complemented and revised by CHS, RH and HL. CHS drafted the English version of the ISFT manual. JS is first author of the original ISFT manual, and CHS is first author of the original publication of our pilot RCT. All authors critically reviewed this publication for important intellectual content. All authors contributed to the article and approved the submitted version.

## Funding

This project was the winner of the 2014 research competition of the German Association for Systemic Therapy, Counseling and Family Therapy (DGSF). It gained further financial support from the Systemic Society (SG), the Heidehof Foundation, the Institute of Medical Psychology at the University Hospital Heidelberg, and the Center for Psychological Psychotherapy at the Heidelberg University.

## Conflict of interest

The authors declare that the research was conducted in the absence of any commercial or financial relationships that could be construed as a potential conflict of interest.

## Publisher’s note

All claims expressed in this article are solely those of the authors and do not necessarily represent those of their affiliated organizations, or those of the publisher, the editors and the reviewers. Any product that may be evaluated in this article, or claim that may be made by its manufacturer, is not guaranteed or endorsed by the publisher.
